# Inhibition of TGF-β1 by eNOS gene transfer provides cardiac protection after myocardial infarction

**DOI:** 10.1016/S1674-8301(10)60023-1

**Published:** 2010-03

**Authors:** Wei Qin, Xin Chen, Peisheng Liu

**Affiliations:** Department of Cardiothoracic Surgery, Nanjing First Hospital Affiliated to Nanjing Medical University, Nanjing, Jiangsu 210006, China

**Keywords:** endothelial nitric oxide synthase gene, myocardial infarction, cardiac function, cardiomyocyte apoptosis, collagen deposition, transforming growth factor-β_1_

## Abstract

**Objective:**

Endothelial nitric oxide synthase (eNOS) and nitric oxide (NO) have been implicated in protection against myocardial ischemia injury. This study was designed to explore a new method of therapy for myocardial injury by eNOS gene transfection.

**Methods:**

A rat model of myocardial infarction (MI) was established by left anterior descending (LAD) coronary artery ligation. eNOS gene in an adenovirus vector was delivered locally into the rat heart and hemodynamic parameters were examined after 3 weeks, Matrix metalloproteinase-2 and 9 (MMP-2, MMP-9) mRNA were measured by reverse transcription polymerase chain reaction (RT-PCR), and the protein levels of eNOS, caspase-3, and transforming grouth factor β_1_ (TGF-β_1_) were determined by western blot assay.

**Results:**

eNOS gene transfer significantly reduced cardiomyocyte apoptosis and improved cardiac function. In addition, eNOS significantly reduced the mRNA levels of MMP-2 and MMP-9. In the eNOS gene transfected group, the activation of caspase-3 and TGF-β_1_ were decreased. However, the protection was reversed by administration of the NOS inhibitor, N(ω)-nitro-l-arginine methyl ester (L-NAME).

**Conclusion:**

These results demonstrate that the eNOS provides cardiac protection after myocardial infarction injury through inhibition of cardiac apoptosis and collagen deposition, and suppression of TGF-β_1_.

## INTRODUCTION

Cardiac remodeling after myocardial infarction (MI) involves myocyte hypertrophy, chamber dilation, and interstitial fibrosis. Alterations in both cardiomyocytes and collagen matrix lead to contractile dysfunction and contribute to the progression of ventricular enlargement and heart failure[1]–[2]. Endothelial nitric oxide synthase (eNOS) is a major source of nitric oxide (NO), which plays an important role in the pathophysiology of postinfarction ventricular remodeling and heart failure[3]. NO regulates vascular tone and cardiomyocyte contractility and protects against myocyte apoptosis and hypertrophy[4]–[5]. Inhibition of eNOS production results in impaired endothelium-dependent vasodilation and vascular endothelial growth factor (VEGF)-induced mobilization of endothelial progenitor cells. Furthermore, eNOS-deficient mice develop more severe cardiac dysfunction after MI than wild-type mice[6], whereas endothelial over-expression of eNOS has been shown to attenuate cardiac dysfunction in mice after MI[7]. Therefore, some drugs that specifically upregulate eNOS may be used for improving ventricular remodeling and function after MI[8].

Based on the above, we increased the expression of eNOS through exogenous eNOS gene transfection into rat heart and then evaluated the post-MI results, thereby exploring a new method of therapy for MI.

## MATERIALS AND METHODS

### MI model and gene transfection

Sprague-Dawley rats from the laboratory animal center of Nanjng Medical University (Nanjng, China) were used and maintained under a controlled environment of 20-22°C, 12/12 h light/dark cycle, and 50-70% humidity, with food and water available ad libitum. All experiments requiring the use of animals received prior approval from Nanjng Medical University and were performed according to USDA-approved protocols. MI was surgically induced in male Sprague-Dawley rats weighting 230-250g by ligation of the left anterior descending coronary artery as described elswhere[9].

In brief, rats were anesthetized (ketamine hydrochloride, 70-85 mg/kg, ip), intubated, and mechanically ventilated before surgery. An electrocardiogram (ECG) monitor was then connected. The chest was opened via an anterior thoracotomy in the third or fourth intercostal space to expose the heart, and the heart was rapidly exteriorized. The proximal left anterior descending coronary artery was ligated about 2 mm distal to its aortic origin with a 5-0 silk suture. The heart was then returned to its original position, and the incision was closed. Acute myocardial ischemia was deemed successful on the basis of regional cyanosis of the myocardial surface distal to the suture, accompanied by elevation of the ECG ST segment.

Half an hour after the operation, 60 MI rats were randomly divided into three groups. In the transfection control group (Ad.CMV-lacZ) and the eNOS transfection (Ad.CMV-eNOS) group, the appropriate solution [2×10^10^ pfu/ml in phosphate-buffered saline (PBS)] in a volume of 200 µl was injected intramyocardially with a curved 27-gauge needle into five to seven sites near the MI area at the anterior left ventricle (LV) wall. The first group (*n* = 20) was injected with Ad.CMV-lacZ and the second group (*n* = 20) received Ad.CMV-eNOS (Shanghai, Freegene Corporation, China), and a third group (*n* = 20) was injected with Ad.CMV-eNOS followed by N(ω)-nitro-l-arginine methyl ester (L-NAME) administration (35 mg/kg iv, 30 min after LAD coronary artery ligation and 12 h before sacrifice). In addition, sham-operated animals (*n* = 10) underwent identical surgery except for the coronary artery ligation and were used for the control group.

### Detection of hemodynamic parameters [10]

Three weeks after the operation, all the rats were anesthetized as described above. A micromanometer-tipped catheter (Millar Instruments, USA) was inserted into the right carotid artery and then advanced into the LV. Mean arterial pressure (MAP), left ventricular end-diastolic pressure (LVEDP), and ± LV dP/dt were recorded and analyzed by a polygraph system (Physiology Lab, Nanjing Medical University).

### Analysis of gene expression by RT-PCR

Total RNA was extracted with Trizol reagent (Gibco, USA) according to the manufacturer's instructions and quantitated by absorbance at 260 nm. The reaction volume of the reverse transcription polymerase chain reaction (RT-PCR) was 25 µl. Reaction temperature was set at 42 °C for 30 min. Primer sequences are shown in ***Table 1***. β-actin was used as the internal standard. PCR amplification was carried out in a volume of 25 µl containing 2 µl of the template, 2.5 µl PCR buffer (10×) and 0.2 µl Taq polymerase, 2.5 mmol/L of each dNTP (TaKaRa, 2.5 mmol/L), 1.5 µl MgCl_2_ (25 mmol/L), 1 µl primers (Promega Corp., USA; 10 µmol/L, forward and reverse). Cycling parameters: one cycle of 5 min at 95°C; 32 cycles of 30 s at 95°C, 30 s at 57∼60°C and 30 s at 72°C; and a final extension at 72°C for 7 min, then preserve at 4°C

**Table 1:** Primer sequences used

primer	Sequence (5′ to 3′)	Size(bp)
MMP-2	AGGACAAGTGGTCCGCGTAAAG	510
	CCACTTCCGGTCATCATCGTAGT	
MMP-9	GGGAACGTATCTGGAAATTCG	520
	CAGAACCGACCCTACAAAGTTG	
β-actin	ATATCGCTGCGCTCGTCGTC	760
	GCATCGGAACCGCTCATTGC	

### Western blot analysis

Myocardial cytosolic proteins were separated by sodium dodecyl sulfate polyacrylamide gel electrophoresis and transferred to a nitrocellulose membrane ((ECL-Hybond-nitrocellulose membranes, Amersham, USA). The membrane was blocked with TBS-T (1*TBS, 0.3% Tween 20) containing 5% dry milk and incubated with primary antibody overnight at 4°C. After three washes with TBS-T, the membrane was blocked and incubated with secondary antibody for 2 hours at room temperature. The primary antibodies used included anti-eNOS and anti-caspase-3 (Cell Signaling Technology, USA), and anti-TGF-β_1_ (Santa Cruz Biotechnology, USA). β-actin was employed as the internal control. The secondary antibody was an HRP-conjugated goat anti-Rabbit IgG.

### Statistical analysis

All data, except survival rates, are expressed as mean ± SD and differences among the experimental group means were tested for using one-way analysis of variance followed by Fisher's protected least significant difference (PLSD). Survival analysis was performed by the Kaplan-Meier method, and between-group differences in survival were tested by the log-rank test. Values of *P* < 0.05 were considered significant.

## RESULTS

### Survival rate

**Fig. 1:**
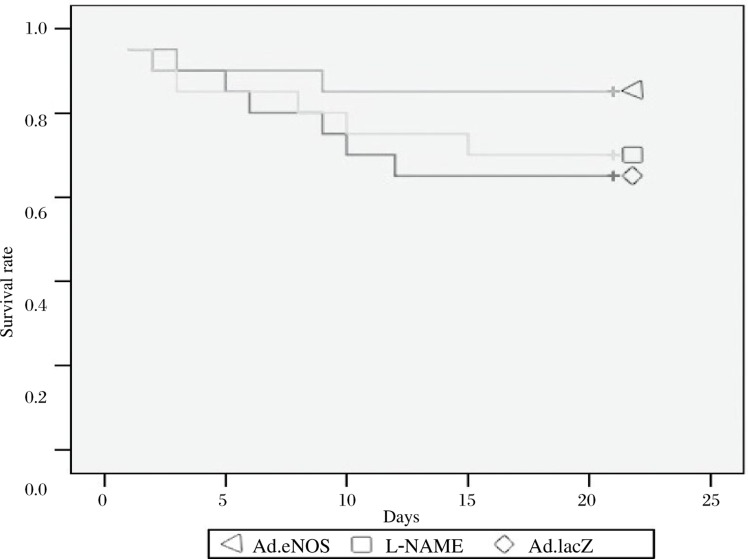
The survival rate of each group at different times.

No rat died from LV rupture, lung infection, pneumothorax, or hemorrhage. There were no deaths in the sham-operated groups (survival rate : 100%). The survival rate of Ad.eNOS group was significantly higher compared to Ad.lacZ group and L-NAME group at each time point. The survival rate of the L-NAME group was significantly lower than the Ad.eNOS group. (***Table 2*** ***and*** ***Fig. 1***)

**Table 2:** The survival rate of each group

	Sham(*n* = 10)	Ad.lacZ(*n* = 20)	Ad.eNOS(*n* = 20)	L-NAME(*n* = 20)
1 week	100(10/10)	80(16/20)	90(18/20)	85(17/20)
2 week	100(10/10)	65(13/20)	85(17/20)	75(15/20)
3 week	100(10/10)	65(13/20)	85(17/20)	70(14/20)

(%)

### Cardiac function

Hemodynamic studies at 3 weeks demonstrated that Ad.eNOS significantly attenuated LV dysfunction (***Table 3***). In comparison with the sham group, the other three groups showed a significant decrease in MAP (105.3 ± 7.2 vs 78.1 ± 6.8, 89.6 ± 6.1, and 83.4 ± 6.5 respectively, *P* < 0.05); Meanwhile, eNOS partially improved systolic function (Ad.eNOS group *vs* Ad.lacZ group: 89.6±6.1 *vs* 78.1±6.8, *P* < 0.05). LVEDP was significantly increased in MI rats, and was attenuated in Ad.eNOS group animals (Ad.eNOS group *vs* Ad.lacZ group: 8.2±0.7 *vs* 16.5±1.3, *P* < 0.05). Characteristic impairments in contractility (+dP/dt) and diastolic function (-dP/dt) after MI significantly improved after eNOS delivery, and the effects were reversed by L-NAME administration (+dP/dt: L-NAME group *vs* Ad.eNOS group: 2023.0±99.8 *vs* 2396.4±103.2, *P* < 0.05; -dP/dt: L-NAME group vs Ad.eNOS group: 1817.5±81.9 *vs* 2104.9±86.4, *P* < 0.05).

**Table 3:** Hemodynamic parameters at 3 weeks after operation

	Sham(*n* = 10)	Ad.lacZ(*n* = 13)	Ad.eNOS(*n* = 17)	L-NAME(*n* = 14)
MAP(mmHg)	105.3 ± 7.2	78.1 ± 6.8	89.6 ± 6.1*	83.4 ± 6.5
LVEDP(mmHg)	1.8 ± 0.2	16.5 ± 1.3	8.2 ± 0.7*	12.3 ± 0.5
+ dp/dt(mmHg/s)	4129.4 ± 95.1	1817.8 ± 100.7	2396.4 ± 103.2*	2023.0 ± 99.8
- dp/dt(mmHg/s)	3673.1 ± 78.2	1734.2 ± 87.9	2104.9 ± 86.4*	1817.5 ± 81.9

+ dP/dt: maximum first derivative of pressure; -dP/dt: minimum first derivative of pressure. *P < 0.05 vs other MI groups

(means ± SD)

### eNOS gene delivery increases eNOS expression

**Fig. 2:**
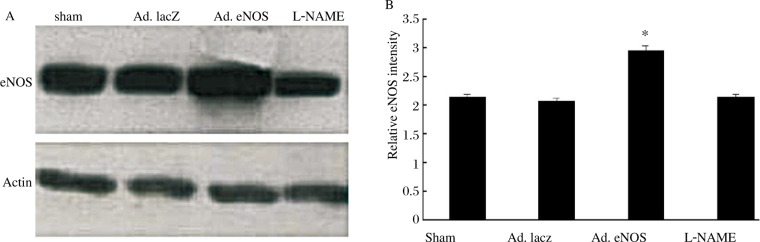
Identification of eNOS formation in the rat left ventricle at 3 weeks after MI. A: western blot analysis showing eNOS expression. B: Quantitative analysis of eNOS / Actin, *P < 0.05 means Ad.eNOS vs other MI groups.

Using an intramyocardial injection strategy, we delivered Ad.eNOS locally into the left ventricle of the rat MI model. Three weeks after the operation, expression of eNOS was identified by western blot analysis of the LV, as shown in ***Fig. 2***. Ad.eNOS delivery increased eNOS levels significantly compared to the sham and Ad.lacZ transfer group (Ad.eNOS group vs sham group, Ad.lacZ group: 2.97±0.08 *vs* 2.14±0.03, 2.08±0.04 respectively, *P* < 0.05). Furthermore, these effects were blocked by L-NAME (L-NAME group *vs* Ad.eNOS group: 2.13±0.04 *vs* 2.97±0.08, *P* < 0.05), indicating that successful local Ad.eNOS delivery increased eNOS expression levels in the infarcted myocardium.

### eNOS reduces the mRNA lever of matrix metalloproteinase (MMP)-2 and MMP-9

**Fig. 3:**
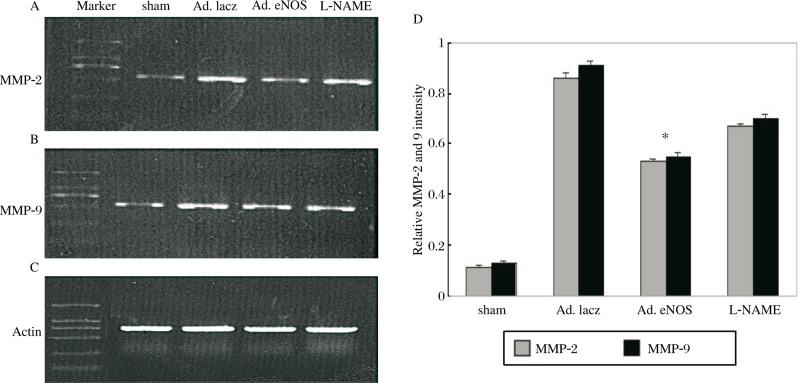
RT-PCR showing the levels of MMP-2 (A), MMP-9 (B), and Actin (C) mRNA in each group, D: Quantitative analysis of relative intensity of MMP-2 and MMP-9, *P < 0.05 means Ad.eNOS vs other MI groups.

RT-PCR was performed to determine the mRNA expression of MMP-2 and MMP-9 in the LV myocardium, which was used to evaluate the early protective role of eNOS after MI. The results (***Fig. 3***) demonstrated that their mRNA expressions were significantly lower in the Ad.eNOS group than in the Ad.lacZ and L-NAME groups (MMP-2: Ad.eNOS group vs Ad.lacZ group and L-NAME group: 0.53±0.01 vs 0.86±0.03 and 0.67±0.01, respectively, *P* < 0.05; MMP-9: Ad.eNOS group vs Ad.lacZ group and L-NAME group: 0.55±0.03 vs 0.91±0.03 and 0.70±0.03, respectively, *P* < 0.05), indicating that eNOS may decrease MMP-2 and MMP-9 in the infarcted myocardium. Interestingly, the mRNA level of MMP-2 and MMP-9 in the L-NAME group was higher than in the Ad.eNOS group, and we inferred that these effects were blocked by L-NAME.

### eNOS inhibits the expression of caspase-3

**Fig. 4:**
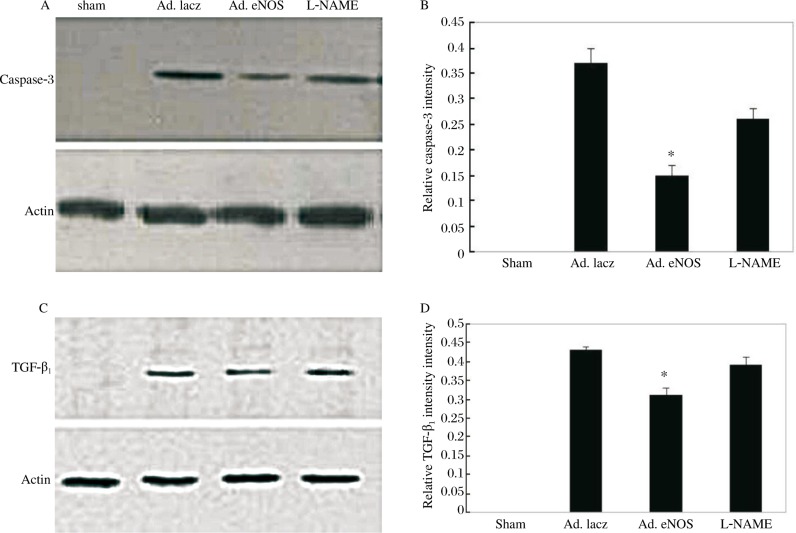
Expression of Caspase-3 (A, B) and TGF-β_1_ (C, D) in the rat left ventricle at 3 weeks after MI. A: western blot analysis showing Caspase-3 level. B: Quantitative analysis of Caspase-3 / Actin, *P < 0.05 means Ad.eNOS vs other MI groups. C: western blot analysis showing TGF-β_1_ level. D: Quantitative analysis of TGF-β_1_ / Actin, *P < 0.05 means Ad.eNOS vs other MI groups.

To evaluate the apoptosis after MI, we examined the caspase-3 expression after eNOS gene delivery as shown in ***Fig. 4A and 4B***. In comparison with sham rats, the expression of caspase-3 was significantly increased in MI rats. Western blot analysis showed that eNOS gene transfer significantly reduced caspase-3 levels compared with the Ad.lacZ group (0.15±0.03 *vs* 0.37±0.04 respectively, *P* < 0.05). Furthermore, this effect was also reversed by L-NAME (0.26±0.03 *vs* 0.15±0.03, *P* < 0.05).

### Effects of eNOS on transforming grouth ofactor β_1_TGF-β_1_) signaling

We measured TGF-β_1_ expression levels in the infarcted LV using western blot analysis. TGF-β_1_, which was virtually non-existent in the sham group was significantly activated after MI, with or without eNOS gene injection. The TGF-β_1_ level was reduced significantly by eNOS transfection (Ad.eNOS group *vs* Ad.lacZ group: 0.31±0.02 vs 0.43±0.01, *P* < 0.05), whereas L-NAME reversed this effect (L-NAME group *vs* Ad.eNOS group: 0.39±0.02 vs 0.31±0.02, *P* < 0.05) (***Fig. 4C and 4D***)

## DISCUSSION

Left ventricular (LV) remodeling after MI is the major cause of heart failure. Some drugs that specifically upregulate eNOS are designed to limit ventricular remodeling after MI, and can decrease the incidence of congestive heart failure and improve survival, although the mechanisms of the protection process have not been fully elucidated. There are 3 isoforms of eNOS: neuronal NOS (nNOS), inducible NOS (iNOS), and endothelial NOS (eNOS)[11]. The eNOS-derived NO serves to promote vascular homeostasis and might affect cardiac myocyte function, modulating many processes contributing to LV performance, including ventricular compliance, myocardial overload compensation, and ischemic injury[12]. Moreover, several studies[13],[14] have shown that increased NO formation resulted in a reduction in the inflammatory response and reduced the LV dysfunction. Previous studies[6],[7] have shown that cardiac remodeling after MI was attenuated in transgenic mice overexpressing eNOS, but worsened in mice deficient in the eNOS gene (eNOS KO mice). Similarly, pressure overload-induced LV remodeling was exacerbated in eNOS KO mice [15],[16]. Based on these previous findings, the pace of exploration in gene therapy has been accelerated.

Cardiomyocyte-specific eNOS overexpression improved LV performance and remodeling after infarction, suggesting that strategies to increase eNOS-derived NO production might provide promising treatments to improve LV remodeling and function in the failing heart [17]–[20]. In the present study, we showed that the ventricular remodeling process after MI was associated with increased eNOS production from the non-infarcted myocardium and demonstrated that eNOS gene transfection attenuated the remodeling and preserved cardiac function in a rat model of MI. We thought that using local eNOS gene delivery, eNOS-derived NO contributed to those beneficial effects through inhibition of cardiomyocyte apoptosis, as well as reducing the degradation of matrix in the rat MI model. The survival rate of the Ad.eNOS group was significantly higher than the Ad.lacZ group and L-NAME group at each point time. Meanwhile, eNOS partially improved systolic and diastolic function, but these effects were blocked by L-NAME.

MMPs have been found to play a major role in normal physiologic tissue remodeling, inflammation and tumor spread. Active forms of MMP-2 and MMP-9 play a pivotal role in collagen accumulation and degradation processes. The study of Ikonomidis *et al*[21] showed that they were up-regulated after MI. In this study, their transcription levels were significantly increased after MI in comparison with sham operation. Furthermore, in the Ad.eNOS group, the high expression of eNOS decreased the mRNA levels of MMP-2 and MMP-9. However, this effect was blocked by the inhibitor of eNOS (L-NAME). These results suggested that the increased expression of eNOS prevents the LV collagen metabolic disorder and protects cardiac function.

We also examined the caspase-3 expression after eNOS gene delivery to evaluate the apoptosis in MI rats. Recent studies have demonstrated that myocyte apoptosis at the site of infarction as well as in the surviving unaffected areas, rather than the acute loss of myocytes by necrosis in the infarcted myocardium, is the major causative factor that contributes to the development of post-MI LV remodeling[22],[23]. In our study MI up-regulated the protein expression of caspase-3, while increasing eNOS expression down-regulated the caspase-3 level. The results suggested that the extent of myocyte apoptosis was reduced by eNOS gene delivery.

The above discussion provides evidence that inhibition of cardiomyocyte apoptosis and collagen metabolic disorder by eNOS delivery after MI could contribute to the prevention of post-MI cardiac dysfunction. However, we still do not know the mechanism of eNOS gene therapy in MI rats, so we measured TGF-β_1_ levels in the infarcted myocardium. TGF-β is a locally generated cytokine that has been implicated as a major stimulator of tissue fibrosis[24]. It has a major influence on fibroblast proliferation and extracellular matrix (ECM) production, particularly of collagen and fibronectin, while reducing degradation of these components[25],[26]. TGF-β expression has been shown to increase not only during cardiac hypertrophy, but also in post-MI hearts[27],[28]. These findings lead to the hypothesis that TGF-β may adversely affect LV remodeling and failure after MI. On the contrary, induction of TGF-β expression as well as supplementation with exogenous TGF-β could protect cardiomyocytes against ischemia injury[29],[30].In our experiment, we found improvement of cardiac function and LV remodeling by eNOS gene therapy, associated with down-regulation of TGF-β_1_ expression after MI injury. Taken together, we favor the hypothesis that eNOS may play a protective role by modulating TGF-β_1_. The study of Chen *et al*[31] also suggested that TGF-β_1_ attenuated alterations in NOS and protein kinase B phosphorylation in myocytes exposed to hypoxia-reoxygenation.

In this study, we chose the adenovirus as the gene therapy vehicle. There are some advantages in using an adenoviral vector over other approaches for gene transfer. The advantages include the high efficiency for infecting target cells, infecting not only proliferating cells but also nonproliferating cells, accommodating very large DNA inserts, being grown to high titers, and extrachromosomal replication to obviate the hazard of insertional mutugenesis. But there are still some important issues in gene therapy, such as the time window of gene transfection, the effectiveness, and gene expression and regulation after gene delivery.

## SUMMARY

Local eNOS gene delivery significantly reduced cardiac fibrosis and myocardial apoptosis, and protected cardiac function after MI. The effects of eNOS were associated with suppression of TGF-β_1_ signaling.
